# DADApy: Distance-based analysis of data-manifolds in Python

**DOI:** 10.1016/j.patter.2022.100589

**Published:** 2022-09-19

**Authors:** Aldo Glielmo, Iuri Macocco, Diego Doimo, Matteo Carli, Claudio Zeni, Romina Wild, Maria d’Errico, Alex Rodriguez, Alessandro Laio

**Affiliations:** 1 International School for Advanced Studies (SISSA), Via Bonomea 265, Trieste, Italy; 2 Banca d’Italia, Italy; 3 Functional Genomics Center, ETH Zurich/UZH, Winterthurerstrasse 190, Zurich, Switzerland; 4 Swiss Institute of Bioinformatics, Quartier Sorge – Batiment, Amphipole 1015, Lausanne, Switzerland; 5 The Abdus Salam International Centre for Theoretical Physics (ICTP), Strada Costiera 11, Trieste, Italy

**Keywords:** manifold analysis, intrinsic dimension, density estimation, density-based clustering, metric learning, feature selection

## Abstract

DADApy is a Python software package for analyzing and characterizing high-dimensional data manifolds. It provides methods for estimating the intrinsic dimension and the probability density, for performing density-based clustering, and for comparing different distance metrics. We review the main functionalities of the package and exemplify its usage in a synthetic dataset and in a real-world application. DADApy is freely available under the open-source Apache 2.0 license.

## Highlights

• DADApy is a Python software library to characterize data manifolds
• DADApy can compute intrinsic dimension, density, cluster structures, and optimal metrics
• DADApy is not based on projections and can work also on topologically complex manifolds
• DADApy has an easy-to-use Python interface and efficient C-compiled routines

## The bigger picture

Data are often represented via many thousands of features. Fortunately, in most applications, such high-dimensional spaces are very sparsely populated, and data points effectively live on low-dimensional “data manifolds.” This is the key reason behind the success of dimensionality reduction schemes, which, however, cannot be easily deployed on data manifolds with nontrivial geometries and topologies, where a set of coordinates capable of describing the manifold globally cannot exist. In these scenarios, one can analyze the data manifold directly, without an explicit dimensional reduction step, and compute fundamental properties, such as the intrinsic dimension of the manifold and the density of the points lying on it. DADApy implements a set of methods recently developed to this aim. DADApy is easy-to-use as it is written entirely in Python, but also computationally efficient as time-consuming routines are C-compiled through Cython.

## Graphical abstract

**Figure:**
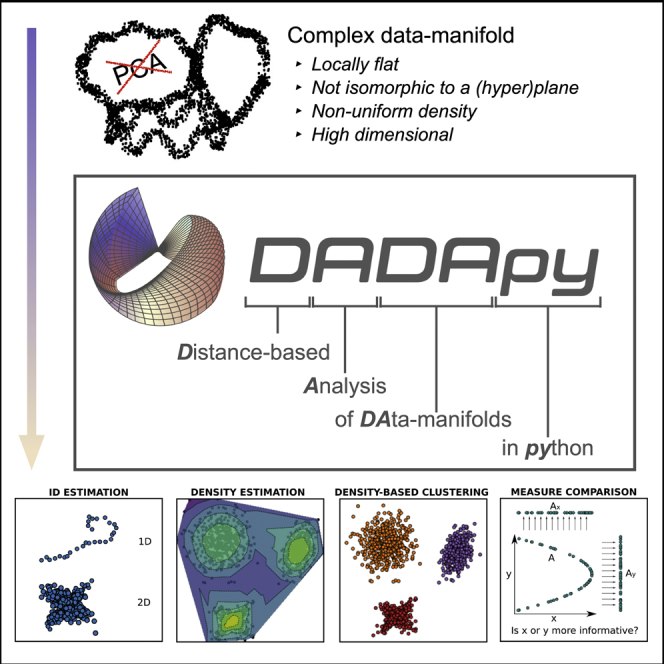

## Teaser

Real-world data are typically represented by high-dimensional features, but live on low-dimensional data manifolds with a great deal of hidden structure. One can analyze such a structure, for instance, by estimating the intrinsic dimension of the manifold, as well as the density of the points lying on it. DADApy collects several algorithms for data manifolds characterization that have already proven effective in specific applications, aims to popularize them, and to make them available for data-science practitioners.

## Introduction

The need to analyze large volumes of data is rapidly becoming ubiquitous in all branches of computational science, from quantum chemistry, biophysics, and materials science^1^^,^^2^ to astrophysics and particle physics.^3^

**Figure 1:**
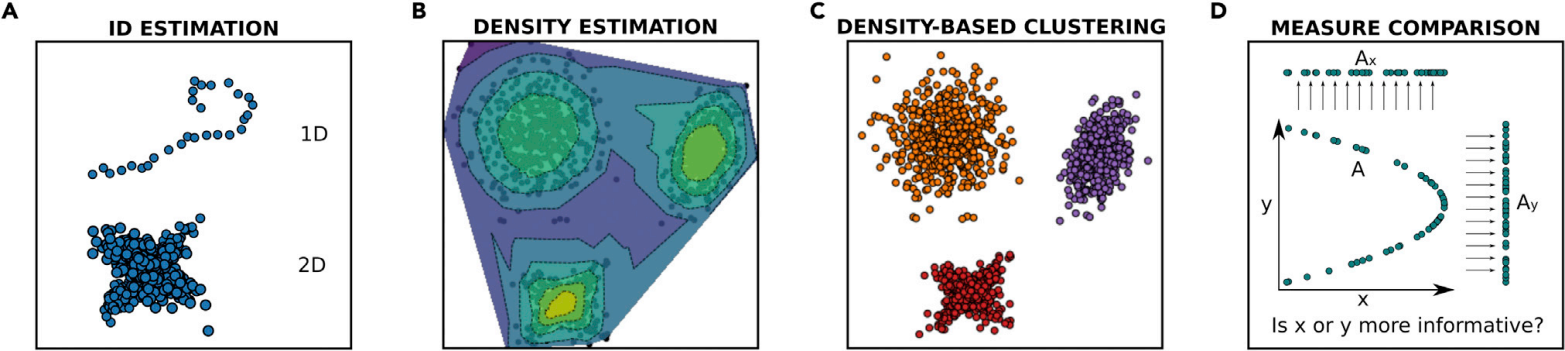
An illustration of the four main classes of tasks that DADApy can perform From (A) to (D): Intrinsic dimension estimation, density estimation, density peaks estimation (i.e., density-based clustering), and comparison of distance measures.

In many practical applications, data come in the form of a large matrix of features, and one can think of a dataset as a cloud of points living in the very high-dimensional space defined by these features. The number of features for each data point can easily exceed the thousands, and if such a cloud of points were to occupy the entire space uniformly, there would be no hope of extracting any kind of usable information from data.^4^^,^^5^ Luckily this never happens in practice, and real-world datasets possess a great deal of hidden intrinsic structure. The most important one is that the feature space, even if very high dimensional, is very sparsely populated. In fact, the points typically lie on a data manifold of much lower dimension than the number of features of the dataset (Figure 1A). A second important hidden structure, which is almost ubiquitous in real-world data, is that the density of points on such a manifold is far from uniform (Figure 1B). The data points are instead often grouped in density peaks (DPs) (Figures 1B and 1C), at times well separated from each other, at times organized hierarchically in “mountain chains.”

DADApy implements in a single and user-friendly software a set of state-of-the-art algorithms to characterize and analyze the intrinsic manifold of a dataset. In particular, DADApy implements algorithms aimed at estimating the intrinsic dimension (ID) of the manifold (Figure 1A) and the probability density of the data (Figure 1B), at inferring the topography and the relative position of the DPs by density-based clustering (Figure 1C) and, finally, at comparing different metrics, finding in this way the features that are better suited to describe the manifold (Figure 1D).

All these approaches belong to the class of unsupervised learning methods and are designed to work also in situations in which only the distances between data points are available instead of their features. Therefore, the same tools can be used for analyzing a molecular dynamics trajectory (where features are available) but also a metagenomics or a linguistic database, where one can only define a similarity or a distance between the data.

Another important feature of the methods included in the package is that they are specifically designed to work even when the ID of the data manifold is relatively high, of order ten or more, and if the manifold is topologically complex, and, in particular, not isomorphic to a hyperplane. Therefore, the package can be considered complementary to other packages, such as Scikit-learn,^6^ which implement classical approaches for unsupervised manifold learning, which should be preferred in simpler cases, such as PCA,^7^ kernel-PCA,^8^ or Isomap.^9^

In the following, we first briefly describe the four classes of algorithms implemented in DADApy. We then illustrate the structure of the package and demonstrate its usage for the analysis of both a synthetic and a realistic dataset. We will also discuss the computational efficiency of the implementations, demonstrating that the package can be used to analyze datasets of $10^{6}$ points or more, even with moderate computational resources.

## Results and discussion

### Description of the methods

#### ID estimators

The ID of a dataset can be defined as the minimum number of coordinates that are needed to describe the data manifold without significant information loss.^10^^,^^11^ In our package we provide the implementation of a class of approaches that are suitable to estimate the ID using only the distances between the points, and not the features. Most of these approaches are rooted in the observation that, in a uniform distribution of points, the ratio $\mu_{i}$ of the distances of two consecutive nearest neighbors of a point *i* are distributed with a Pareto distribution, which depends only on the ID. This allows defining a simple likelihood for the *N* observations of $\mu_{i}$, one for each point of the dataset:

(Equation 1) $$p\left(\left\{\mu_{i}\right\}|\text{ID}\right)=\prod_{i=1}^{N}\text{ID}\;\mu_{i}^{-\left(\text{ID}+1\right)}.$$

The ID is then estimated either by maximizing the likelihood,^12^ by Bayesian inference,^13^ or by linear regression after a suitable variable transformation.^14^ We refer to these estimators as two nearest neighbors (2NN) estimators.

It is possible that the data manifold possesses different IDs depending on the scale of variations considered. For example, a spiral dataset can be one-dimensional on a short scale, but two-dimensional on a larger scale. Hence, one might be interested in computing an ID estimate as a function of the scale. The package provides two routines to perform this task. The first method allows to probe the ID at increasing length scales by sub-sampling the original dataset. By virtue of the reduced number of points considered, the average distance between them will be larger; this can be then interpreted as the length scale at which the ID is computed. Obviously, subsampling the dataset also increases the variance of the ID estimate. The second method, an algorithm called "generalized ratios ID estimator (Gride),” circumvents this issue by generalizing the likelihood in Equation 1 to directly probe longer length scales without subsampling.^13^

After using one of these algorithms, one can select the ID of the dataset as the estimate that is most consistently found across different scales. However, this choice is often not straightforward, and for a more in depth discussion on this topic we refer to Denti et al.^13^^,^^14^ and Facco et al.^13^^,^^14^

ID estimation has been successfully deployed in a number of applications, ranging from the analysis of deep neural networks,^15^ to physical applications, such as phase transition detection^16^ and molecular force-field validation.^17^

#### Density estimators

The goal of density estimation is to reconstruct the probability density ρ(x) from which the dataset has been harvested. The package implements a non-parametric density estimator called point-adaptive *kNN* (PA*k*),^18^ which uses as input only the distances between points and, importantly, is designed to work under the explicit assumption that the data are contained in an embedding manifold of relatively small dimension. This algorithm is an extension of the standard *k*NN estimator,^19^ which estimates the density on a point as proportional to the empirical density sampled in its immediate surrounding. More precisely, the *k*NN estimates can be written as

(Equation 2) $$\rho_{i}=\frac{1}{N}\frac{k}{V_{i,k}},$$

where *k* is the number of nearest neighbors considered, and $V_{i,k}$ is the volume they occupy. The volume is typically computed as $V_{i,k}=\omega_{\text{ID}}d_{i,k}^{\text{ID}}$, where $\omega_{\text{ID}}$ is the volume of unit sphere in $R^{\text{ID}}$ and $d_{i,k}$ is the distance between point *i* and its *k*th nearest neighbor.

In PA*k* the number of neighbors *k* used for estimating the density around point *i* is chosen adaptively for each data point by an unsupervised statistical approach in such a way that the density, up to that neighbor, can be considered approximately constant. This trick dramatically improves the performance of the estimator in complex scenarios, where the density varies significantly at short distances.^18^ Importantly, the volumes that enter the definition of the estimator are measured in the low-dimensional intrinsic manifold rather than in the full embedding space. This prevents the positional information of the data from being diluted on irrelevant directions orthogonal to the data manifold. Assuming that the data manifold is Riemannian, namely locally flat, it can be locally approximated by its tangent hyperplane and distances between neighbors, the only distances used in the estimator, can be measured in this low-dimensional Euclidean space. This allows to operate on the intrinsic manifold without any explicit parametrization. The only prerequisite is an estimate of the local ID, since this is needed to measure the volumes directly on the manifold.

Another key difference between *k*NN and PA*k* estimators is that *k*NN assumes the density to be exactly constant in the neighborhood of each point, while PA*k* possesses an additional free parameter that allows to describe small density variations. The PA*k* density estimator can be used to reconstruct free energy surfaces, especially in high-dimensional spaces,^18^^,^^20^, ^21^, ^22^ and it can also be used for a detailed analysis of the data, as in Offei-Danso et al.,^23^ where a distinct analysis of the data points with different densities lead to some physical insight about the system under study.

The same estimator can be used also for estimating the density on points that do not belong to the dataset,^24^ a procedure that has been recently used to quantify the degree to which test data are well represented by a training dataset.^25^

Finally, PA*k* is commonly used within the density-based clustering algorithms discussed in the following section.

#### DP clustering

The different “peaks” of the probability density can be considered a natural partition of the dataset into separate groups or “clusters.” This is the key idea underlying density peak (DP) clustering,^26^ implemented in DADApy. This algorithms works by first estimating the density $\rho_{i}$ of all points *i*, for example using the PA*k* method described in the previous section. Then, the minimum distance $\delta_{i}$ between point *i* and any other point with higher density is computed as

(Equation 3) $$\delta_{i}=\underset{j\;|\;\rho_{j}>\;\rho_{i}}{\text{min}}d_{ij}.$$

The peaks of the density (and hence the cluster centers) are expected to have both a high density $\rho_{i}$ and a large distance $\delta_{i}$ from points with higher density, and are hence selected as the few points for which both $\rho_{i}$ and $\delta_{i}$ are very large. The selection is typically done by plotting $\rho_{i}$ against $\delta_{i}$ and visually identifying the outliers of the distribution. Once the cluster centers are found, each remaining point is assigned to the same cluster as its nearest neighbor of higher density.

In DP clustering the DPs must be specified by the user, and this arbitrariness represents an obvious source of errors. The advanced DP (ADP) clustering approach,^27^ also available in DADApy, proposes a solution to this problem. In ADP clustering, all local maxima of the density are initially considered DPs, and a statistical significance analysis of each peak is subsequently performed. A peak *c* is considered statistically significant only if the difference between the log density of the peak $\ln\;\rho_{c}$ and the log density of any neighboring saddle point $\ln\;\rho_{cc^{\text{′}}}$ is sufficiently larger than the sum of the errors on the two estimated quantities

(Equation 4) $$\ln\;\rho_{c}-\ln\;\rho_{cc^{\text{′}}}>Z\left(\sigma_{c}+\sigma_{cc^{\text{′}}}\right).$$

If this is not the case, the two peaks *c* and $c^{\text{′}}$ are merged into a single peak. This process is iterated until no peak that is not statistically significant is remaining. The parameter *Z* appearing in Equation 4 can be interpreted as the statistical significance threshold of the found peaks. A higher value of *Z* will give rise to a smaller number of peaks with a higher statistical significance. Typical values range from 1 to 5. ADP and DP are general clustering tools, and as such have been used in different fields, including single-cell transcriptomics,^28^^,^^29^ spike-sorting,^30^^,^^31^ word embedding,^32^ climate modelling,^33^ Markov state modelling,^34^ and the analysis of molecular dynamics simulations,^35^^,^^36^ just to mention some of them.

Another clustering algorithm available in DADApy is *k*-peaks clustering.^37^ In short, this method is a variant of ADP that takes advantage of the observation that the optimal $k_{i}$ is high in two cases: (1) in high-density regions, due to the high concentration of points, and (2) in vast regions where the density is everywhere constant. Therefore, the peaks in $k_{i}$ correspond either to peaks in density or to the center of large regions with nearly constant density (e.g., metastable states stabilized by entropy). An example application of *k-*peaks clustering can be found in Sormani et al.,^37^ where it was used to describe the free-energy landscape of the folding/unfolding process of a protein.

#### Metric comparisons

In several applications, the similarity (or the distance) between different data points can be measured using very different metrics. For instance, a group of atoms or molecules in a physical system can be represented by their Cartesian coordinates, by the set of their inter-particle distances, or by a set of dihedral angles, and one can measure the distance between two configurations with any arbitrary subset of these coordinates. Similarly, the “distance” between two patients can be measured taking into account their clinical history, any subset of blood exams, radiomics features, genome expression measures, or a combination of those.

It might hence be useful to evaluate the relationships between all these different manners to measure the similarity between data points. DADApy implements two methods for doing this: the neighborhood overlap and the information imbalance. Both approaches use only the distances between the data points as input, making the approaches applicable also when the features are not explicitly defined (e.g., a social network, a set of protein sequences, a dataset of sentences).

The neighborhood overlap is a simple measure of equivalence between two representations.^38^ Given two representations *a* and *b*, one can define two *k*-adjacency matrices $A_{ij}^{a}$ and $A_{ij}^{b}$ as matrices of dimension N×N, which are all zero except when *j* is one of the *k* nearest neighbors of point *i*. The neighborhood overlap χ(a,b) is then defined as

(Equation 5) $$\chi \left(a\text{,}\;b\right)=\frac{1}{N}\sum_{i}\frac{1}{k}\sum_{j}A_{ij}^{a}A_{ij}^{b}.$$

Note that the term $A_{ij}^{a}A_{ij}^{b}$ is equal to one only if *j* is within the *k* nearest neighbors of *i* both in *a* and in *b*, otherwise it is zero. For this reason, the neighborhood overlap can also be given a very intuitive interpretation: it is the average fraction of common neighbors in the two representations. If χ(a,b)=1 the two representations can be considered effectively equivalent, while if χ(a,b)=0 they can be considered completely independent. The parameter *k* can be adjusted to improve the robustness of the estimate but in practice this does not significantly change the results obtained as long as k≪N.^38^

In the original article,^38^ the neighborhood overlap was proposed to compare layer representations of deep neural networks and to analyze in this their inner workings.

The information imbalance is a recently introduced quantity capable of assessing the information that a distance measure *a* provides about a second distance measure *b*.^39^ It can be used to detect not only whether two distance measures are equivalent or not, but also whether one distance measure is more informative than the other. The information imbalance definition is closely linked to information theory and the theory of copula variables.^39^ However, for the scope of this article it can be empirically defined as

(Equation 6) $$\begin{array}{l} \text{Δ}\left(a\to b\right)=\frac{2}{N}\left\langle r^{b}|r^{a}=1\right\rangle \\ \;=\frac{2}{N^{2}}\sum_{i,j:\;r_{ij}^{a}=1}r_{ij}^{b} \end{array},$$

where $r_{ij}^{a}$ is the rank matrix of the distance *a* between the points (namely $r_{ij}^{a}=1$ if *j* is the nearest neighbor of *i*, $r_{ij}^{a}=2$ if *j* is the second neighbor, and so on). In words, the information imbalance from *a* to *b* is proportional to the empirical expectation of the distance ranks in *b* conditioned on the fact that the distance rank between the same two points in *a* is equal to one. If Δ(a→b)≈0 then *a* can be used to describe *b* with no loss of information.

When measuring the information imbalances between two representations we can have three scenarios. If Δ(a→b)≈Δ(b→a)≈0 the two representations are equivalent, if Δ(a→b)≈Δ(b→a)≈1 the two representations are independent, and, finally, if Δ(a→b)≈0 and Δ(b→a)≈1 we have that *a* is informative about *b* but not vice versa, therefore *a* is more informative than *b*. The information imbalance allows for effective dimensional reduction since a small subset of features that are the most relevant, either for the full set or for a target property, can be identified and selected.^39^ This feature selection operation is available in DADApy and can be performed as a pre-processing step before the tools described in the previous sections are deployed.

The information imbalance proved successful in dealing with atomistic and molecular descriptors, either to directly perform compression^39^ or to quantify the information loss incurred by competing compression schemes.^40^ In the original article,^39^ the information imbalance was also proposed for detecting causality in time series—with illustrative results shown on COVID-19 time series—and to analyze or optimize the layer representations of deep neural networks.

### Software structure and usage

**Figure 2:**
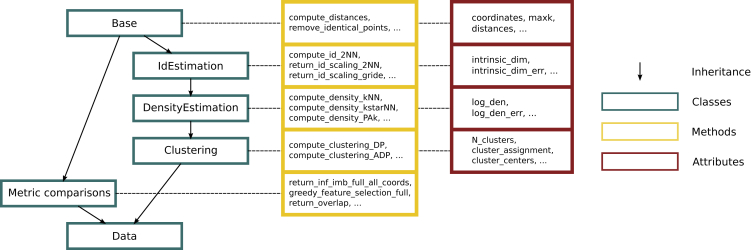
The class structure of the package Classes are highlighted in blue boxes, and the main methods and attributes of each class are reported in the yellow and red boxes, respectively. Relationships of inheritance are indicated as black arrows. The class Data inherits from all other classes, thus providing easy access to all available algorithms of the package.

DADApy is written entirely in Python, with the most computationally intensive methods being sped up through Cython. It is organized in six main classes: Base, IdEstimation, DensityEstimation, Clustering, MetricComparison, and Data. The relationships of inheritance between these classes, as well as the main methods and attributes available in each class are summarized in Figure 2. The Base class contains basic methods of data cleaning and manipulation that are inherited in all other classes. Attributes containing the coordinates and/or the distances defining the dataset are contained here. Then, in a train of inheritance: IdEstimation inherits from Base; DensityEstimation inherits from IdEstimation and Clustering inherits from DensityEstimation. Each of these classes contains as methods the algorithms described in the previous section, under the same name. The inheritance structure of these classes is well motivated by the fact that, to perform a density-based clustering one first needs to compute the density, and to perform a density estimation one first needs to know the ID, which can be estimated only if the distances are preliminarily computed. The MetricComparison class contains the algorithms described in the section titled “Metric comparisons” used to compare couples of representations using the distances between points.

The class Data does not implement any extra attribute or method but, importantly, it inherits all methods and attributes from the other classes. As such, Data provides easy access to all available algorithms of the package and is the main class that is used in practice.

**Figure 3:**
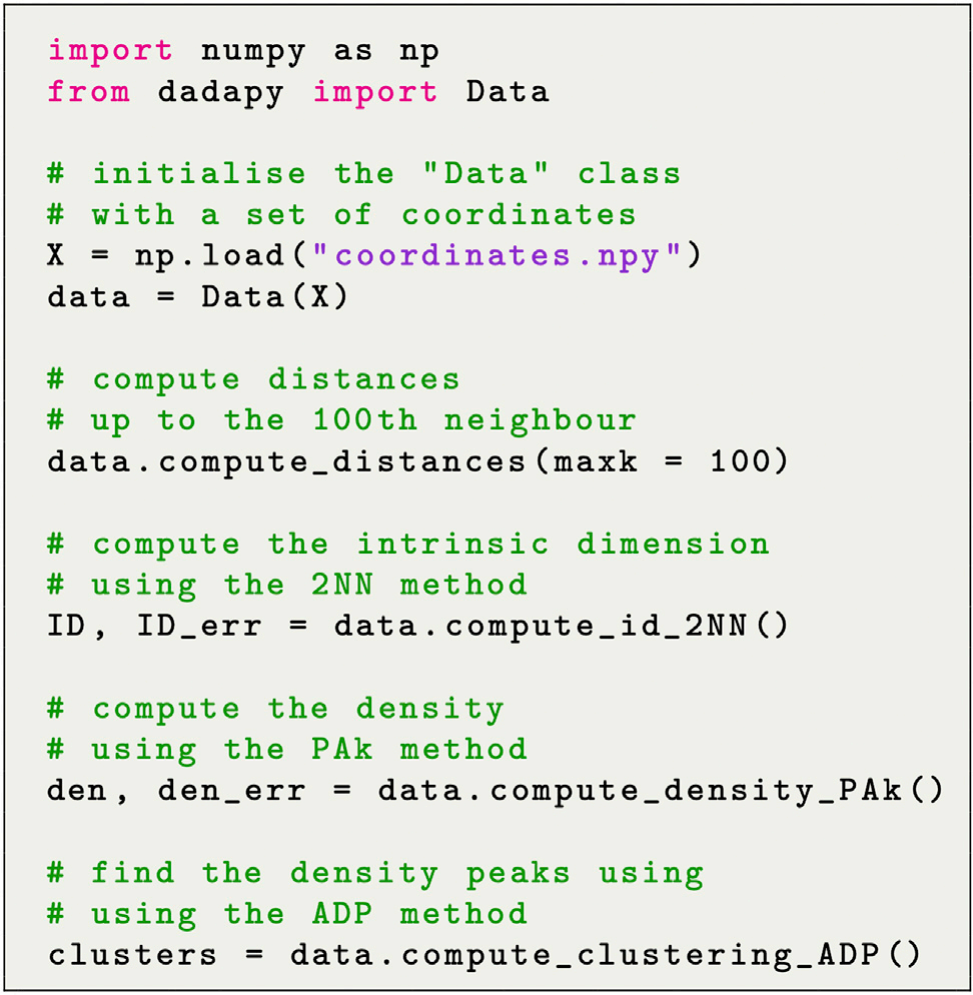
A simple DADApy script

A typical usage of DADApy is reported in Figure 3. In this simple example a Data object is first initialized with the matrix containing the coordinates of the points shown in Figures 1B and 1C, and later a series of methods are called sequentially to compute the distances, the ID, the density (Figure 1B), and finally the DPs (clusters) of the dataset (Figure 1C). In the example given, Data is initialized with a matrix of coordinates, and the distances between points are later computed. Note that, however, the object could have been equivalently initialized directly with the distances between points, and all methods in the package would work equivalently. This is particularly important for those applications for which coordinates are not available, but distances can be computed, such as DNA or protein sequences, or networks.

The main aim of the package is to provide user-friendly, fast, and light routines to extract some of the most common and fundamental characteristics of a data manifold through solid statistical and numerical techniques. DADApy offers high-speed code with reduced memory consumption. These features are achieved by exploiting locality. In particular, it is generally enough to compute the distances between each point and a small number of its neighbors (defined in DADApy by an attribute named maxk), and hence such distances can be computed and stored with close-to-linear time and memory requirements.

We believe that the Python interface of DADApy will encourage its rapid diffusion, as Python is by far the most used language in the computational science community nowadays. We are aware that Python is, however, a notoriously inefficient language for large-scale computation. In DADApy we circumvent this shortcoming by implementing all the heavy numerical routines using Cython extensions, which essentially generate C-compilable code that runs with very high efficiency (typically over two orders of magnitude faster in evaluation time than the pure Python implementation). In this manner we are able to maintain the user friendliness of Python without sacrificing the computational efficiency of a fully compiled language.

**Figure 4:**
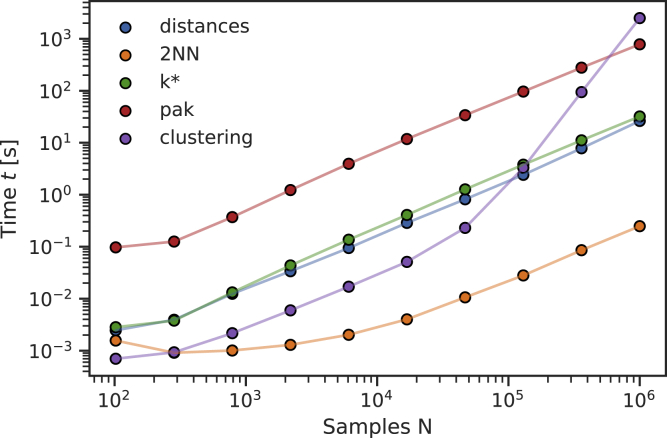
Time complexity of DADApy The time required by the various routines of DADApy grows linearly with the number of samples *N*, with the only exception of ADP (see text for details). The dataset used was two dimensional and we set maxk = 100. The benchmark was performed on an ordinary desktop using a single Intel Xeon(R) CPU E5-2650 v2 at 2.60 GHz.

All of the mentioned properties allow to easily analyze up to a million points on an ordinary laptop within minutes. This can be seen in Figure 4, where we report the time spent by the code on many DADApy routines as a function of the number *N* of points of the dataset, using a neighborhood of maxk = 100 points. The plot shows that all methods scale linearly in computational time with *N*, with the exception of the ADP clustering, whose scaling becomes unfavorable for more than 50,000 points. This is a consequence of the neighborhood size maxk being much smaller than the number of points *N* of the dataset, a condition which forces the estimation of many fictitious DPs that take a long time to be merged together. The problem can be solved by appropriately increasing maxk when necessary.

The runtime performance for the computation of the distances also scales linearly with the embedding dimension *D*, while the other routines take as input the computed distances, and are thus independent on *D*. Therefore, when *D* is very large, say $D⪆10^{4}$, the distance computation can represent the actual computational bottleneck of the package.

The code has been thoroughly commented and documented through a set of easy-to-run Jupyter notebooks, an online manual, and an extensive code reference. This can allow new users approaching DADApy to quickly learn to use it, as well as to modify or extend it.

### Illustration on a topologically complex synthetic dataset

**Figure 5:**
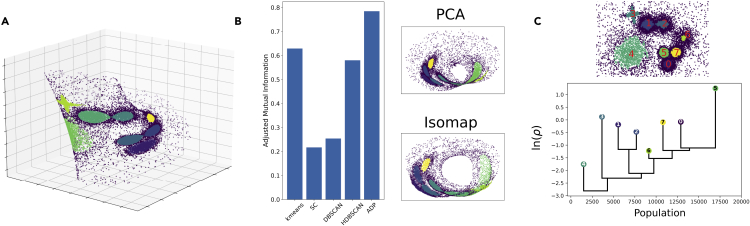
Example usage of DADApy for the analysis of a topologically complex synthetic dataset (A) The dataset analyzed, consisting of clusters lying on a 2D sheet twisted to form a Möbius strip and immersed in a noisy 50D space. (B) The accuracy of some common clustering methods on reconstructing the original clusters (in order: Kmeans, Spectral Clustering [SC], DBSCAN, HDBSCAN, and ADP), as well as two low-dimensional projections. (C) Summary of the results obtained using 2NN ID estimation, PA*k* density estimation and ADP clustering. The top part shows the estimated density peaks, while the bottom part shows the dendrogram of the dataset. The y axis of the dendrogram reports the log density of the density peaks and of the saddle points. The x axis provides an indication on the relative cluster sizes, since each cluster is in the middle of a region proportional to its population. This region is delimited by the links in which these clusters are involved and, in the case of the first and last clusters, by the beginning and end of the graph.

We now illustrate the use of some key DADApy methods on the synthetic dataset depicted in Figure 5A, and consisting of a 2D plane with eight clusters, twisted to form a 3D Möbius strip and finally embedded in a noisy 50D space. The reference 2D dataset is taken from d’Errico et al.,^27^ and consists of data points sampled from an analytic density function, with points belonging to a single mode of this density assigned to the same cluster, and all other considered unassigned.

Despite the 2D inner structure of the dataset, common projection methods can easily fail as a consequence of the nontrivial topological properties of the data manifold. This is illustrated in Figure 5B, where PCA and ISOMAP projections are reported.

One key advantage of the methods implemented in DADApy is their ability to exploit the low-dimensional structure of the data without any explicit projection. In this case, for example, we compute the ID using the Gride method (see “intrinsic dimension estimators"), which is correctly identified around 2. We then use the ID to provide accurate density estimates using the PA*k* method from “density estimators,” and finally identify the clusters (or DPs) using the ADP algorithm from “density peak clustering.” The end result is a cluster assignment that is remarkably close to the ground truth, and often superior to other state-of-the-art clustering schemes that do not exploit the low-dimensional structure of the data (see Figure 5B).

Another unique feature of DADApy is the ability of compactly representing the cluster structure through a special kind of dendrogram reporting the log densities of the DPs and of the saddle points between them. The bottom part of Figure 5C depicts the dendrogram for the Möbius strip data, which can be seen to provide a remarkably accurate perspective of the relationship between the estimated DPs shown in the upper panel of the figure.

Note that the dendrogram can be generated independently of the ID of the manifold, unlike most graphical data representations which are practically limited to three dimensions, thus providing a robust way to visualize the cluster structure even for the common scenario of ID>3 manifolds.

The Jupyter notebook used to perform the analysis described in this section can be found at https://github.com/sissa-data-science/DADApy/blob/main/examples/notebook_mobius.ipynb.

### Usage for a realistic application

**Figure 6:**
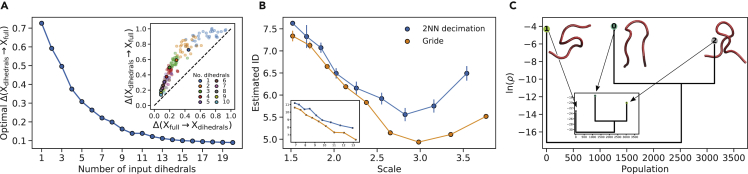
Example usage of DADApy for the analysis of a biomolecular trajectory (A) The computation of the information imbalance between a compact molecular representation $X_{\text{dihedrals}}$ (optimally selected sets of dihedral angles with increasing size) and a much higher dimensional one $X_{\text{full}}$ (the full space of heavy atom distances). The inset shows the information imbalance between the space of heavy atom distances and the space of dihedral angles, and vice versa. For clarity, the depicted points are sparsed out. (B) The computation of the intrinsic dimension across different scales using both 2NN and Gride. The main graph refers to the space of 15 dihedrals, while the inset refers to the space of 4,278 heavy atom distances. (C) A dendrogram visualization of the peaks and the saddle points of the density, estimated using PA*k* and the ADP clustering algorithm. Peptide backbones of cluster center structures are drawn next to their corresponding peaks. The main graph refers to the space of dihedrals, while the inset refers to the space of heavy atom distances. In both cases, the central and rightmost peaks capture the main macro states of the peptide and are much more populated than the leftmost peak. The two cluster assignments are identical for roughly 90% of the data points.

We now exemplify and showcase the usage of DADApy for the analysis of a biomolecular trajectory. The dataset is composed of 41,580 frames from a replica-exchange MD simulation (400 ns, 340 K replica, dt = 2 fs) of the 10-residue peptide CLN025, which folds into a beta hairpin.^41^ Several numerical representations are possible for this trajectory. A very high-dimensional one is given by the set of all distances between the heavy atoms, which amounts to 4,278 features. Such a representation is possibly very redundant, and in fact typically more compact representations are used to describe systems of this type. For example, a compact representation for this system can be taken as the set of all its 32 dihedral angles.^42^^,^^43^ In Figure 6A we use DADApy to compute the information imbalance from the space of heavy atom distances to the space of the dihedral angles for an increasing number of dihedral angles, and vice versa. Not surprisingly, the compact space of dihedral angles is seen to be almost equally informative to the very high-dimensional heavy atom distance space, with information imbalance $\text{Δ}\left(X_{\text{dihedrals}}\to X_{\text{full}}\right)$ lower than 0.1 when considering around 15 angles (Figure 6A). We thus select the set of the 15 most informative dihedral angles as the collective variables to represent this dataset, since the information imbalance reaches a plateau around this number.

We then use DADApy to compute the ID of the dataset along different scales through both decimation and the Gride algorithm^13^ (Figure 6B). The two procedures provide fairly overlapping estimates for the ID, which is comprised between 5 and 8 within short range distances, and thus much lower than the original feature space. We continue by estimating the density through the PA*k* algorithm, for which we set the ID to 7. This ID selection is motivated by the observation that the density is a local property computed at short scales but, importantly, selecting a lower ID consistent with Figure 6B (say, 5 or 6) does not significantly affect the results. Finally, we use DADApy to perform clustering using the ADP algorithm. The results are shown in Figure 6C.

ADP clustering (Z = 4.5) produces three clusters. The biggest cluster is the folded beta hairpin state of the protein, as depicted in Figure 6C (cluster 0). A cluster of roughly half the size is made of a collapsed twisted loop structure (Figure 6C, cluster 2). Since CLN025 is suspected to have two main metastable states, the folded hairpin and a denatured collapsed state,^44^ we suggest that the twisted loop could be the dominant topology of the denatured collapsed ensemble. The high occurrence of the twisted loop might be due to the simulation temperature of 340 K, which is just below the experimental melting temperature of CLN025 of 343 K.^45^ Less than 1% of the structures are in cluster 1, which is composed of denatured extended and less-structured topologies.

The 32-dimensional space of dihedrals used so far in our analysis is known to be well suited to differentiate meaningful protein structures but, to showcase the possibility of using DADApy to work in very-high-dimensional spaces, we performed a similar analysis also on the 4,278-dimensional space of all heavy atom distances. Using this alternative data description we performed ID estimation with the 2NN method, density estimation with the PA*k* estimator, and clustering with the ADP algorithm (ID = 9; Z = 3.5). The resulting dendrogram is shown as an inset of Figure 6C.

As clear from the figure, we find a remarkably similar cluster structure, defined by the two major macrostates of the molecule, the beta pin and the twisted loop, as well as the cluster with unstructured configurations.

The equivalence in the two cluster assignments is confirmed by the fact that 89% of the data points are assigned to the same cluster independently of the data representation.

A Jupyter notebook containing the analyses performed in this section is available at https://github.com/sissa-data-science/DADApy/blob/main/examples/notebook_beta_hairpin.ipynb along with the necessary datasets.

### Conclusions

In this work we introduce DADApy, a software package for quickly extracting fundamental properties of data manifolds. DADApy is written entirely in Python, which makes it easy to use and to extend; and it exploits Cython extensions and algorithms for sparse computation and sparse memory handling, which make it computationally efficient and scalable to large datasets. The package is documented by a set of easy-to-run Jupyter notebooks and by a code-reference and manual available online.

DADApy includes state-of-the-art algorithms for ID estimation, density estimation, density-based clustering, and distance comparison, which found numerous applications in recent years, but have not yet found widespread usability. We believe this was, at least in part, precisely due to the lack of a fast and easy-to-use software like DADApy, and we hope that our work will allow a growing number of practitioners from different research domains to approach the field of manifold learning.

The algorithms included in DADApy do not rely on low-dimensional projections or on any strong assumptions on the structure of the data. This can be a great advantage, as it makes DADApy suited to analyze topologically complex data manifolds, but it also means that DADApy cannot be used to build low-dimensional maps for data visualization. Other shortcomings of the software are in its level of maturity for industrial-grade standards—DADApy is still a young software—and in the relatively small number of algorithms implemented in it.

We plan to improve DADApy by addressing both of these issues. On the one hand we are working on the development of algorithms that extend many of the methods discussed here, including ID estimators for discrete spaces,^46^ density estimators that exploit data correlations, and more refined feature selection schemes based on the information imbalance, and intend to implement these as new DADApy methods. On the other hand we intend to improve code quality in a variety of directions, such as by increasing unit test coverage, expanding documentation and lint checks, and adding static type checking. Finally, we will greatly welcome open-source contributions to the project.

## Experimental procedures

### Resource availability

#### Lead contact

Further information and requests for resources should be directed to and will be fulfilled by the lead contact, Aldo Glielmo (aldo.glielmo@bancaditalia.it).

#### Materials availability

This study did not generate new materials.

## Data Availability

DADApy is available at https://github.com/sissa-data-science/DADApy (https://doi.org/10.5281/zenodo.6998360), and the notebooks to generate the key graphs of Figures 5 and 6 are available at https://github.com/sissa-data-science/DADApy/blob/main/examples/notebook_mobius.ipynb and https://github.com/sissa-data-science/DADApy/blob/main/examples/notebook_beta_hairpin.ipynb, respectively. We strongly encourage the scientific community to fork the repository, submit pull requests, and open new issues through the GitHub interface.

## References

[1] Schütt K.T., Chmiela S., von Lilienfeld O.A., Tkatchenko A., Tsuda K., Müller K.-R. (2020). Machine learning meets quantum physics. Lect. Notes Phys..

[2] Glielmo A., Husic B.E., Rodriguez A., Clementi C., Noé F., Laio A. (2021). Unsupervised learning methods for molecular simulation data. Chem. Rev..

[3] Carleo G., Cirac I., Cranmer K., Daudet L., Schuld M., Tishby N., Vogt-Maranto L., Zdeborová L. (2019). Machine learning and the physical sciences. Rev. Mod. Phys..

[4] Keogh E., Mueen A. (2010).

[5] Aggarwal C.C., Hinneburg A., Keim D.A. (2001). International conference on database theory.

[6] Pedregosa F., Varoquaux G., Gramfort A., Michel V., Thirion B., Grisel O., Blondel M., Prettenhofer P., Weiss R., Dubourg V. (2011). Scikit-learn: machine learning in Python. J. Mach. Learn. Res..

[7] Abdi H., Williams L.J. (2010). Principal component analysis. WIREs. Comp. Stat..

[8] Schölkopf B., Smola A., Müller K.-R. (1997). International conference on artificial neural networks.

[9] Balasubramanian M., Schwartz E.L. (2002). The isomap algorithm and topological stability. Science.

[10] Campadelli P., Casiraghi E., Ceruti C., Rozza A. (2015). Intrinsic dimension estimation: relevant techniques and a benchmark framework. Math. Probl Eng..

[11] Camastra F., Staiano A. (2016). Intrinsic dimension estimation: Advances and open problems. Inf. Sci..

[12] Levina E., Bickel P., Saul L., Weiss Y., Bottou L. (2004). https://proceedings.neurips.cc/paper/2004/file/74934548253bcab8490ebd74afed7031-Paper.pdf.

[13] Denti F., Doimo D., Laio A., Mira A. (2021). Distributional results for model-based intrinsic dimension estimators. arXiv.

[14] Facco E., d’Errico M., Rodriguez A., Laio A. (2017). Estimating the intrinsic dimension of datasets by a minimal neighborhood information. Sci. Rep..

[15] Ansuini A., Laio A., Macke J.H., Zoccolan D., Wallach H., Larochelle H., Beygelzimer A., d’ Alché-Buc F., Fox E., Garnett R. (2019). https://proceedings.neurips.cc/paper/2019/file/cfcce0621b49c983991ead4c3d4d3b6b-Paper.pdf.

[16] Mendes-Santos T., Turkeshi X., Dalmonte M., Rodriguez A. (2021). Unsupervised learning universal critical behavior via the intrinsic dimension. Phys. Rev. X.

[17] Capelli R., Gardin A., Empereur-Mot C., Doni G., Pavan G.M. (2021). A data-driven dimensionality reduction approach to compare and classify lipid force fields. J. Phys. Chem. B.

[18] Rodriguez A., d’Errico M., Facco E., Laio A. (2018). Computing the free energy without collective variables. J. Chem. Theory Comput..

[19] Loftsgaarden D.O., Quesenberry C.P. (1965). A nonparametric estimate of a multivariate density function. Ann. Math. Statist..

[20] Zhang J., Chen M. (2018). Unfolding hidden barriers by active enhanced sampling. Phys. Rev. Lett..

[21] Marinelli F., Faraldo-Gómez J.D. (2021). Force-correction analysis method for derivation of multidimensional free-energy landscapes from adaptively biased replica simulations. J. Chem. Theory Comput..

[22] Salahub D.R. (2022). Multiscale molecular modelling: from electronic structure to dynamics of nanosystems and beyond. Phys. Chem. Chem. Phys..

[23] Offei-Danso A., Hassanali A., Rodriguez A. (2022). High-dimensional fluctuations in liquid water: Combining chemical intuition with unsupervised learning. J. Chem. Theory Comput..

[24] Carli M., Laio A. (2021). Statistically unbiased free energy estimates from biased simulations. Mol. Phys..

[25] Zeni C., Anelli A., Glielmo A., Rossi K. (2022). Exploring the robust extrapolation of high-dimensional machine learning potentials. Phys. Rev. B.

[26] Rodriguez A., Laio A. (2014). Clustering by fast search and find of density peaks. science.

[27] d’Errico M., Facco E., Laio A., Rodriguez A. (2021). Automatic topography of high-dimensional data sets by non-parametric density peak clustering. Inf. Sci..

[28] Ziegler C.G.K., Allon S.J., Nyquist S.K., Mbano I.M., Miao V.N., Tzouanas C.N., Cao Y., Yousif A.S., Bals J., Hauser B.M. (2020). Sars-cov-2 receptor ace2 is an interferon-stimulated gene in human airway epithelial cells and is detected in specific cell subsets across tissues. Cell.

[29] Habib N., Li Y., Heidenreich M., Swiech L., Avraham-Davidi I., Trombetta J.J., Hession C., Zhang F., Regev A. (2016). Div-seq: single-nucleus rna-seq reveals dynamics of rare adult newborn neurons. Science.

[30] Yger P., Spampinato G.L., Esposito E., Lefebvre B., Deny S., Gardella C., Stimberg M., Jetter F., Zeck G., Picaud S. (2018). A spike sorting toolbox for up to thousands of electrodes validated with ground truth recordings in vitro and in vivo. Elife.

[31] Sperry Z.J., Na K., Jun J., Madden L.R., Socha A., Yoon E., Seymour J.P., Bruns T.M. (2021). High-density neural recordings from feline sacral dorsal root ganglia with thin-film array. J. Neural. Eng..

[32] Wang W.M., Liu J.C., Xu J., Tian G., Liu C.-L., Hao H. (2016). Semantic expansion using word embedding clustering and convolutional neural network for improving short text classification. Asian Pac. J. Trop. Med..

[33] Margazoglou G., Grafke T., Laio A., Lucarini V. (2021). Dynamical landscape and multistability of a climate model. Proc. Math. Phys. Eng. Sci..

[34] Pinamonti G., Paul F., Noé F., Rodriguez A., Bussi G. (2019). The mechanism of rna base fraying: molecular dynamics simulations analyzed with core-set Markov state models. J. Chem. Phys..

[35] Jong K., Hassanali A.A. (2018). A data science approach to understanding water networks around biomolecules: the case of tri-alanine in liquid water. J. Phys. Chem. B.

[36] Carli M., Sormani G., Rodriguez A., Laio A. (2020). Candidate binding sites for allosteric inhibition of the SARS-CoV-2 main protease from the analysis of large-scale molecular dynamics simulations. J. Phys. Chem. Lett..

[37] Sormani G., Rodriguez A., Laio A. (2020). Explicit characterization of the free-energy landscape of a protein in the space of all its cα carbons. J. Chem. Theory Comput..

[38] Doimo D., Glielmo A., Ansuini A., Laio A., Larochelle H., Ranzato M., Hadsell R., Balcan M.F., Lin H. (2020). Adv. Neural Inf. Process. Syst..

[39] Glielmo A., Zeni C., Cheng B., Csányi G., Laio A. (04 2022). Ranking the information content of distance measures. PNAS Nexus.

[40] Darby J.P., Kermode J.R., Csányi G. (2021). Compressing local atomic neighbourhood descriptors. arXiv.

[41] Honda S., Yamasaki K., Sawada Y., Morii H. (2004). 10 residue folded peptide designed by segment statistics. Structure.

[42] Bonomi M., Branduardi D., Bussi G., Camilloni C., Provasi D., Raiteri P., Donadio D., Marinelli F., Pietrucci F., Broglia R.A., Parrinello M. (2009). Plumed: a portable plugin for free-energy calculations with molecular dynamics. Comput. Phys. Commun..

[43] Cossio P., Laio A., Pietrucci F. (2011). Which similarity measure is better for analyzing protein structures in a molecular dynamics trajectory?. Phys. Chem. Chem. Phys..

[44] McKiernan K.A., Husic B.E., Pande V.S. (2017). Modeling the mechanism of cln025 beta-hairpin formation. J. Chem. Phys..

[45] Honda S., Akiba T., Kato Y.S., Sawada Y., Sekijima M., Ishimura M., Ooishi A., Watanabe H., Odahara T., Harata K. (2008). Crystal structure of a ten-amino acid protein. J. Am. Chem. Soc..

[46] Macocco I., Glielmo A., Grilli J., Laio A. (2022). Intrinsic dimension estimation for discrete metrics. arXiv.

